# Co-occurring mental and substance use disorders in Australia 2020–2022: Prevalence, patterns, conditional probabilities and correlates in the general population

**DOI:** 10.1177/00048674241284913

**Published:** 2024-10-11

**Authors:** Matthew Sunderland, Joshua Vescovi, Cath Chapman, Vikas Arya, Meredith Harris, Philip Burgess, Christina Marel, Katherine Mills, Andrew Baillie, Maree Teesson, Tim Slade

**Affiliations:** 1 The Matilda Centre for Research in Mental Health and Substance Use, The University of Sydney, Sydney, NSW, Australia; 2 Melbourne School of Population and Global Health, The University of Melbourne, Melbourne, VIC, Australia; 3 The University of Queensland, Brisbane, QLD, Australia; 4 Queensland Centre for Mental Health Research, Brisbane, QLD, Australia; 5 Sydney School of Health Sciences, Faculty of Medicine and Health, The University of Sydney, Sydney, NSW, Australia

**Keywords:** Comorbidity, epidemiology, mood disorders, anxiety disorders, substance use disorders

## Abstract

**Background:**

Previous estimates from 2007 found that co-occurring mental and/or substance use disorders were a pervasive feature of Australia’s mental health. Since that time there have been shifts and improvements in the conceptualisation and incorporation of co-occurring disorders in research and treatment settings. The current study provides up-to-date estimates on the prevalence of co-occurring mental and/or substance use disorders, highlights common patterns of co-occurrence, identifies significant correlates and examines any changes in the extent of co-occurring disorders since 2007.

**Methods:**

Data were from the two Australian National Surveys of Mental Health and Wellbeing conducted in 2020–2022 (*N* = 15,893) and 2007 (*N* = 8841). Descriptive statistics were estimated for the number of co-occurring conditions, correlations and pairwise conditional probabilities. Multinomial logistic and robust Poisson regressions were used to identify significant correlates and compare changes in co-occurring conditions across surveys.

**Results:**

Approximately 46% of people with a mental or substance use disorder in the past 12 months experienced two or more diagnosable conditions. There was little evidence to suggest that the prevalence of co-occurring disorders has changed since 2007 (Prevalence Ratio (PR) = 1.08, 95% CI = 0.98–1.18). Subgroup analysis indicated that those aged 16–24 years were significantly more likely to experience any co-occurrence in 2020–2022 compared with those aged 16–24 years in 2007 (PR = 1.44, 95% CI = 1.17–1.77).

**Conclusions:**

Co-occurring mental and substance use disorders remain endemic in Australia. Indeed, they appear to be increasingly problematic in younger, more recent cohorts. The results suggest that continued effort is needed to develop and implement transdiagnostic interventions that target broad contextual and/or societal factors.

## Introduction

Comorbidity can be defined as the co-occurrence of two or more mental and/or substance use disorders within an individual at any defined time. In the current paper, we focus on co-occurrence of disorders within a 12-month period and refer to mental and substance use disorders as defined by the diagnostic algorithms set out in the *Diagnostic and Statistical Manual of Mental Disorders* (4th ed.; DSM-IV). The co-occurrence of mental and substance use disorders is frequently observed and a well-documented phenomenon (First, 2005). Numerous large-scale epidemiological studies have demonstrated that many individuals with a mental disorder also have at least one other disorder. For instance, the National Comorbidity Survey Replication (NCS-R) in the United States revealed that over 40% of individuals who met the criteria for one 12-month mental disorder also met the criteria for at least one other 12-month mental disorder (Kessler et al., 2005).

In Australia, the 2007 National Survey of Mental Health and Wellbeing (NSMHWB) found that around one in four people with a 12-month mental disorder met criteria for another mental disorder in a different disorder class (e.g. mood, anxiety or substance use disorders) (Teesson et al., 2009). The most prevalent co-occurrence was between anxiety and mood disorders, with over half of those with an anxiety disorder also having a co-occurring mood disorder (Teesson et al., 2009). In addition, the 2007 NSMHWB showed that one in five Australians with substance use disorders also had a mood disorder, and around one in three had an anxiety disorder (Teesson et al., 2009). Co-occurring mental and substance use disorders have been associated with poorer treatment outcomes, leading to severe illness and high levels of health service utilisation compared with those with a single mental or substance use disorder (Kessler et al., 2005; Lai et al., 2015; Teesson et al., 2009). Understanding patterns of co-occurrence is crucial for developing effective responses and health service planning.

Thirteen years have passed since the publication of previous national estimates of comorbidity in the Australian population. In that time, advances in the research of co-occurring conditions have begun to change the epidemiological landscape and how the field conceptualises this co-occurrence, and mental disorders in general, and the potential to better treat it. For example, the Unified Protocol for Transdiagnostic Treatment of Emotional Disorders (UP) was developed with the aim of addressing comorbidity in the broader category of emotional disorders (anxiety and mood disorders), concentrating on the common underlying mechanism of neuroticism (Barlow et al., 2010). Several studies have supported the effectiveness of UP in reducing anxiety and depression symptoms (Sakiris and Berle, 2019; Sauer-Zavala et al., 2020). Other integrated treatments for anxiety and substance use have shown positive results in randomised controlled trials (Stapinski et al., 2021). Moreover, pharmacological treatments for mental disorders have long demonstrated the same kind of non-specificity as transdiagnostic psychological treatments, i.e. similar or the same medications are used to treat a wide range of emotional disorders (Dalgleish et al., 2020). In relation to treatment guidelines, there are several noteworthy examples that address co-occurring conditions, including the publication of the Australian comorbidity treatment guidelines and associated training materials (Marel et al., 2023). The training programme has been demonstrated as effective for improving the identification, knowledge, attitudes and confidence of alcohol and other drug workers in relation to co-occurring mental health conditions (Louie et al., 2021; Marel et al., 2023).

Given the substantial shifts in conceptualising and incorporating co-occurrence of disorders in research and treatment settings, it has become increasingly important to reassess the degree and pattern of co-occurrence in the Australian population and draw comparisons with previous estimates derived in 2007. As a field, we need to understand whether the extent of co-occurrence has changed, whether it has stayed the same or whether patterns have started to shift in new directions that require further investigation. The current study aimed to use the most recent Australian NSMHWB conducted in 2020/2022 to provide up-to-date estimates on the prevalence of co-occurring mental and/or substance use disorders, highlight common patterns of co-occurrence, identify their correlates and compare estimates with those identified previously.

## Method

### Sample

Data were from the 2020–2022 NSMHWB. Details of the sampling framework, complex survey design and administration of the survey are provided in Slade et al. (2024). The current study uses combined data from two cohorts that formed the 2020–2022 NSMHWB. The first cohort provided data over an 8-month period from December 2020 to July 2021, whereas the second cohort provided data over an 11-month period from December 2021 to October 2022. The sample represents all usual residents in Australia aged 16–85 years living in private dwellings in urban and rural areas across all states and territories. Very remote parts of Australia and discrete Aboriginal and Torres Strait Islander communities were not included. There were 15,893 fully responding households, representing a response rate of 52%.

The 2007 NSMHWB was used for comparison purposes to determine whether the degree and burden associated with comorbidity has changed in the intervening 13 years. A total of 8841 households fully responded to the survey representing a 60% response rate. Further details of the 2007 NSMHWB can be found in Slade et al. (2009) and detailed comparisons between the instrumentation and method of the 2007 and 2020–2022 surveys are provided in Slade et al. (2024). The use of the data was approved by the Australian Bureau of Statistics in line with their comprehensive data use and safety rules. Given this is a secondary analysis of publicly available data the current study was exempt from human ethics review.

### Measurement

#### Mental disorders

Diagnostic information on mental disorders was obtained using the World Mental Health Consortium version of the Composite International Diagnostic Interview (WMH-CIDI) (Kessler et al., 2004). This interview has strong psychometric properties and calibrated against a structured clinical interview. To determine the diagnostic status of each disorder in the past 12 months, the criteria prescribed in the Diagnostic and Statistical Manual of the American Psychiatric Association 4^th^ edition (*DSM*-IV) were used. To assess the overall impact of co-occurring diagnoses, the hierarchical exclusion rules that restrict the diagnosis of some conditions depending on the presence of others were not applied consistent with 2007 reports.

Diagnostic variables were created for major depressive disorder (MDD), bipolar disorder, dysthymia, generalised anxiety disorder (GAD), obsessive compulsive disorder (OCD), panic disorder, agoraphobia, social anxiety disorder (SAD), post-traumatic stress disorder (PTSD), any alcohol use disorder (abuse and/or dependence; AUD) and any drug use disorder (abuse and/or dependence; drug use included cannabis, stimulants, sedatives and opioids; DUD). A sum of the number of co-occurring past 12-month diagnoses was generated and categorised for subsequent analysis as no disorder, one disorder only, two disorders, or three or more disorders. For an overall summary of disorder co-occurrence classes, three additional variables were created that collapsed diagnoses of (1) MDD, bipolar and dysthymia into any mood disorder; (2) GAD, OCD, panic disorder, agoraphobia, PTSD and SAD into any anxiety disorder; and (3) alcohol use disorder and any drug use disorder into any substance use disorder.

#### Correlates and service use

Demographic and additional clinical information were obtained as part of the broader survey and used in the current study to examine correlates of disorder co-occurrence. These variables included age, sex at birth, country of birth, employment status, remoteness area (ARIA; Accessibility/Remoteness Index of Australia defined as a measure of relative geographic access to services categorised into five classes: major cities, inner regional, outer regional, remote and very remote), socioeconomic status (Socio-Economic Indexes for Areas [SEIFA]; Index of relative socioeconomic disadvantage quintiles with 1 being most disadvantaged and 5 least disadvantaged), psychological distress, service use of any mental health care and suicidality. Service use was measured as a single variable to determine help seeking for an emotional or behavioural problem in the past 12 months from any mental health care practitioner. Psychological distress was measured using the Kessler psychological distress scale (K10) with previously established cut-point to index high psychological distress and increased probability of a clinical diagnosis (Andrews and Slade, 2001; Kessler et al., 2002). Finally, a single suicidality variable was derived using three questions to determine the presence or absence of previous suicidal ideation, suicidal plans or suicide attempts.

### Statistical analysis

Descriptive analysis of the total sample was used to investigate the prevalence of each broad mental disorder class as well as individual mental disorder diagnoses and the proportion of those with only that diagnosis, the diagnosis plus one other diagnosis (two in total) and the diagnosis plus two or more diagnoses (three or more in total). The pairwise bivariate associations (tetrachoric correlations suitable for binary data) between all mental disorder diagnoses were calculated to determine the pattern of co-occurrence. The pairwise conditional probabilities of each mental disorder were calculated by estimating the probability of one disorder given the presence of a target disorder. For example, the probability of MDD was determined among those with a diagnosis of GAD and similarly the probability of GAD was determined among those with a diagnosis of MDD, and so on.

The impact of co-occurrence on each individual disorder (and across the total sample) was determined by calculating the comorbidity to diagnosis inflation ratio (CDIR) (Miozzo et al., 2021). Briefly, the CDIR is calculated by taking each diagnosis of a given disorder (e.g. MDD) and summing the number of co-occurring disorder dyads observed in that disorder (e.g. MDD and GAD, MDD and AUD, etc.). The total number of dyads is then divided by the total number of diagnoses for that disorder to generate an overall summary of the number of co-occurring conditions expected per diagnosis. For example, a CDIR of 1.22 associated with MDD indicates that there are approximately 1.22 co-occurring disorders per diagnosis of MDD.

Significant correlates were determined by creating a new variable to represent the number of co-occurring diagnoses (e.g. only one diagnosis, two diagnoses and three or more diagnoses) and examining the prevalence of co-occurring conditions among each sociodemographic and clinical subgroup. Multinomial logistic regression analysis was then used to estimate the odds ratios (OR) and 95% confidence intervals (95% CI) for each correlate of interest (adjusting for age and sex at birth), using the group with only one diagnosis as the reference category. All data were weighted based on the Australian general population and variances, standard errors (SE) and confidence intervals were adjusted to account for the complex sampling design of the survey.

Finally, the number of co-occurring disorders experienced by respondents from the 2020–2022 National Survey was directly compared with the number of co-occurring disorders experienced by respondents from the 2007 National Survey. A composite binary variable that comprised either one disorder or two or more disorders (any co-occurrence) was generated among those who experienced at least one disorder in the past 12 months from either survey. Poisson regression with robust standard errors was used to estimate the prevalence ratio (PR) of any co-occurrence across the two surveys. Sex at birth and age interactions with survey year were estimated to determine whether the prevalence of co-occurrence differed across the two surveys depending on whether the respondent was male/female or older/younger.

## Results

### Prevalence of common mental disorders and proportion of co-occurring cases

**Figure 1.**
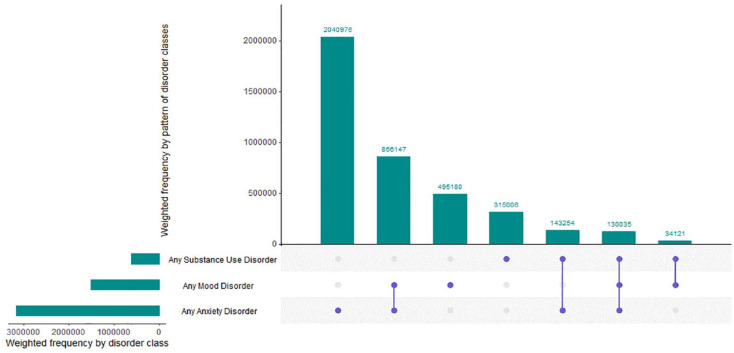
Past 12-month comorbidity profiles for the Australian population.

Table 1 provides the prevalence of each disorder in the general population aged 16–85 years, and among those with a diagnosis, the proportion with only the target disorder, the proportion with one other disorder and the proportion with two or more other disorders. Of note, the proportion of cases with the highest level of co-occurrence was associated with bipolar disorder, agoraphobia, dysthymia, panic disorder, GAD and MDD with between 51% and 81% of the total number of people with these disorders having two or more other disorders. The disorder with the highest proportion of single disorder cases was AUD with 50% of all people with AUD experiencing just AUD in the past 12 months. The weighted frequencies of any mood disorder, any anxiety disorder, and any substance use disorder and the co-occurring patterns across the three classes are provided in Figure 1 (solid dots refer to the presence of a single disorder class and connected dots reflect co-occurring disorder classes). In total, 7.7% (95% CI = 7.2, 8.2) of the population experienced any mood disorder, 16.0% (95% CI = 15.3, 16.8) experienced any anxiety disorder and 3.1% (95% CI = 2.8, 3.5) experienced any substance use disorder in the past 12 months. In terms of broad co-occurrence patterns, those with only anxiety disorders represented the largest disorder class with approximately 2 million Australians or 10.3% (95% CI = 9.7, 10.9), followed by mood and anxiety disorders without substance use disorders accounting for approximately 866,000 Australians or 4.4% (95% CI = 4.0, 4.8).

**Table 1.** Past 12-month prevalence of each mental disorder and proportion of comorbidity in each mental disorder.

Disorder	Total prevalence		Proportion with only one disorder		Proportion with one other disorder		Proportion with two or more other disorders	
	%	95% CI	%	95% CI	%	95% CI	%	95% CI
MDD	7.4	(6.9–8.0)	24.7	(22.2–27.3)	24.1	(20.7–27.9)	51.2	(46.9–55.5)
BP	0.9	(0.7–1.2)	NA	(NA–NA)	NA	(NA–NA)	80.7	(66.7–89.8)
DYS	2.4	(2.1–2.6)	2.8	(1.3–5.7)	19.8	(14.4–26.6)	77.4	(70.5–83.1)
AGO	2.2	(1.9–2.6)	8.8	(5.4–14.1)	11.9	(7.9–17.5)	79.2	(71.8–85.2)
GAD	4.9	(4.4–5.3)	20.3	(17.2–23.8)	20.9	(17.4–24.8)	58.8	(54.4–63.1)
OCD	4.2	(3.9–4.6)	41.7	(36.9–46.7)	20.0	(16.4–24.2)	38.3	(33.2–43.5)
PD	2.7	(2.4–3.1)	19.6	(15.8–24.0)	20.5	(15.9–26.0)	60.0	(53.6–66.0)
SAD	7.7	(7.2–8.4)	34.4	(31.2–37.7)	20.2	(17.1–23.7)	45.4	(41.7–49.2)
PTSD	4.0	(3.6–4.5)	36.9	(31.8–42.3)	21.3	(17.9–25.2)	41.8	(37.0–46.7)
AUD	2.5	(2.2–2.8)	50.2	(42.9–57.6)	16.0	(11.6–21.7)	33.7	(28.3–39.6)
DUD	0.6	(0.5–0.9)	26.6	(19.0–36.0)	32.9	(23.1–44.5)	40.5	(30.2–51.7)

MDD: major depressive disorder; BP: bipolar disorder; DYS: dysthymia; AGO: agoraphobia; GAD: generalised anxiety disorder; OCD: Obsessive compulsive disorder; PD: panic disorder; SAD: social anxiety disorder; PTSD: post-traumatic stress disorder; AUD: any alcohol use disorder; DUD: any drug use disorder.

95% confidence intervals calculated using Wilson’s method. Some cells were suppressed due to Australian Bureau of Statistics (ABS) data safety rules. All diagnostic algorithms were applied without hierarchy rules.

### Pattern of co-occurrence

**Figure 2.**
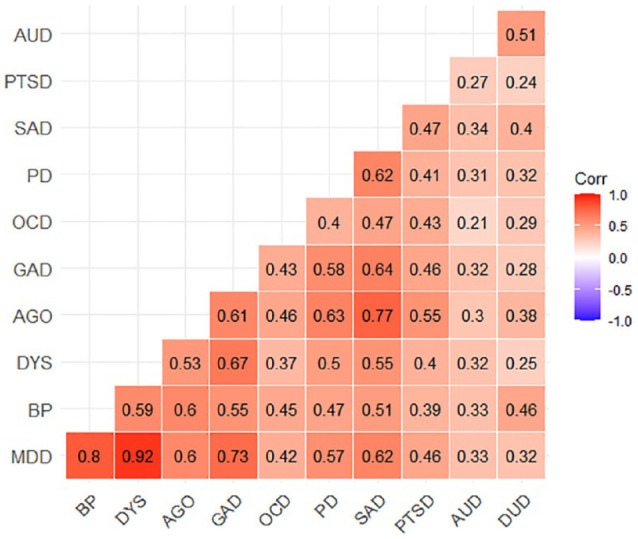
Tetrachoric correlation matrix for past 12-month disorders in the Australian population. AUD: alcohol use disorder; PTSD: post-traumatic stress disorder; SAD: social anxiety disorder; PD: panic disorder; OCD: obsessive compulsive disorder; GAD: generalised anxiety disorder; AGO: agoraphobia; DYS: dysthymia; BP: bipolar disorder; MDD: major depressive disorder.

Figure 2 provides the pairwise tetrachoric correlations between all co-occurring combinations. The mood disorders (MDD, bipolar and dysthymia) demonstrated the strongest pairwise associations with an average correlation of 0.77, followed by the anxiety disorders (agoraphobia, GAD, OCD, panic disorder, SAD and PTSD) with an average correlation of 0.53, followed by substance use disorders with a correlation of 0.51. The smallest pairwise association was between OCD and alcohol use disorder (*r* = 0.21), whereas the largest was associated with dysthymia and MDD (*r* = 0.92). Table 2 provides the pairwise conditional probabilities of mental disorders. The conditional probabilities for each disorder provide some indication of the expected disorder co-occurrence composition in those with a specific diagnosis and these patterns do differ depending on the index condition. For example, the probability of having a diagnosis of social anxiety disorder given the presence of OCD was relatively high at 75%, whereas the probability of having a diagnosis of OCD given the presence of social anxiety disorder was lower at 20%.

**Table 2.** Conditional pairwise probabilities of comorbid 12-month mental disorders.

	MDD (%)	BP (%)	DYS (%)	AGO (%)	GAD (%)	OCD (%)	PD (%)	SAD (%)	PTSD (%)	AUD (%)	DUD (%)
Major depression	–	84.4	93.8	50.8	44.7	52.0	37.9	24.4	25.7	15.7	38.9
Bipolar disorder	11.8	–	10.8	10.1	10.2	13.5	7.0	6.6	5.8	3.4	16.5
Dysthymia	28.9	23.7	–	19.7	18.9	15.0	12.0	9.0	10.7	7.3	7.5
Agoraphobia	18.5	34.3	17.3	–	18.7	26.4	25.2	15.8	21.9	8.2	15.8
Generalised anxiety disorder	32.4	45.8	40.7	30.7	–	33.4	26.2	17.6	19.4	10.7	21.9
Obsessive compulsive disorder	14.5	28.1	17.3	16.4	21.5	–	26.4	19.6	14.7	5.9	20.6
Panic disorder	15.9	25.9	21.9	17.2	26.4	19.1	–	12.9	11.2	9.7	18.5
Social anxiety disorder	39.9	52.6	41.3	43.3	56.4	74.5	34.9	–	31.3	19.3	43.2
Post-traumatic stress disorder	15.4	24.9	20.8	18.3	18.8	36.8	17.8	14.8	–	12.1	8.6
Alcohol use disorder	5.0	7.7	7.6	5.4	8.7	7.3	5.8	3.2	6.4	–	13.6
Drug use disorder	5.4	16.5	3.4	4.8	7.3	6.2	5.7	4.9	2.0	6.0	–

MDD: major depressive disorder; BP: bipolar disorder; DYS: dysthymia; AGO: agoraphobia; GAD: generalised anxiety disorder; OCD: Obsessive compulsive disorder; PD: panic disorder; SAD: social anxiety disorder; PTSD: post-traumatic stress disorder; AUD: any alcohol use disorder; DUD: any drug use disorder.

Probabilities represent the conditional probability of the row given the column, e.g. the probability of having dysthymia given MDD = 29%, whereas the probability of having major depression given DYS = 94%. Some cells are suppressed given ABS data safety rules. All diagnostic algorithms were applied without hierarchy rules.

### Impact of co-occurrence

**Figure 3.**
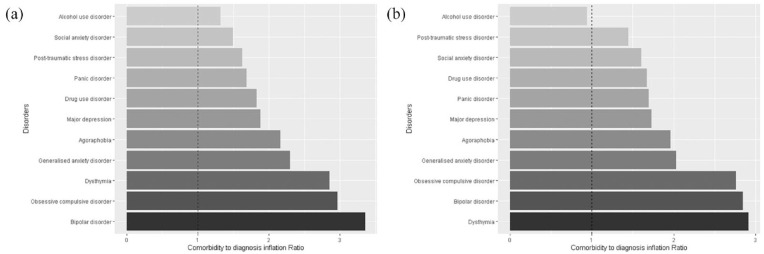
Comorbidity to diagnosis inflation ratios for individual disorders in the (A) 2020–2022 and (B) 2007 National surveys.

The CDIR values for each diagnosis are provided in Figure 3, 2020–2022 estimates in the left panel and 2007 estimates in the right panel. The pervasiveness of co-occurrence is demonstrated by the finding that almost all individual conditions exhibited CDIR values greater than 1, indicating that on average for every disorder there is at least one or more co-occurring disorder. Moreover, the largest individual disorder CDIRs were associated with bipolar disorder, OCD, dysthymia and GAD. These disorders all had a CDIR value > 2.

### Correlates of co-occurrence

Tables 3 and 4 provide percentages and the multinomial logistic regression results associated with demographic and clinical correlates for subgroups of people who have one disorder, two disorders, and three or more disorders. In age and/or sex at birth-adjusted models, respondents aged 16–24 years and 25–44 years demonstrated significantly increased odds of a diagnosis of three or more disorders (compared with one disorder only) compared with those aged 65 + (OR = 2.12 95% CI = 1.52–2.95, OR = 1.61 95% CI = 1.21–2.33). Likewise, being female (OR = 1.45 95% CI = 1.15–1.84), unemployed or not in the labour force (OR = 2.51 95% CI = 1.56–4.06, OR = 1.91 95% CI = 1.43–2.55), born in Australia (OR = 1.62 95% CI = 1.28–2.05), did not complete year 12 (OR = 1.45 95% CI = 1.10–1.90), separated/widowed/divorced or never married (OR = 2.04 95% CI = 1.45–2.87, OR = 2.13 95% CI = 1.64–2.77), a SEIFA quintile of 2 (OR = 1.58 95% CI = 1.12–2.21), high psychological distress (OR = 7.25 95% CI = 5.31–9.90), past 12-month service use (OR = 4.24 95% CI = 3.36–5.35) or past 12-month suicidality (OR = 3.96 95% CI = 2.73–5.74) demonstrated significantly increased odds of three or more disorders in comparison to one disorder only. Being separated/widowed/divorced or never married (OR = 1.46 95% CI = 1.03–2.07, OR = 1.44 95% CI = 1.03–2.01), high psychological distress (OR = 2.05 95% CI = 1.26–3.35), past 12-month service use (OR = 2.55 95% CI = 2.00–3.24) and past 12-month suicidality (OR = 1.87 95% CI = 1.14–3.04) were significantly associated with a diagnosis of two disorders (compared with one disorder only).

**Table 3.** Demographic and clinical correlates of past 12-month comorbidity.

	% with one disorder		% with two disorders		% with three or more disorders	
	%	95% CI	%	95% CI	%	95% CI
Age						
16–24	17.1	(15.3–19.1)	8.4	(6.9–10.3)	10.8	(9.2–12.6)
25–44	12.3	(11.2–13.5)	4.4	(3.8–5.2)	5.9	(5.2–6.8)
45–64	10.2	(9.1–11.5)	3.1	(2.6–3.7)	3.9	(3.3–4.6)
65+	5.8	(4.9–6.8)	1.9	(1.4–2.5)	1.7	(1.4–2.2)
Sex at birth^a^						
Male	10.2	(9.4–11.1)	3.3	(2.8–3.8)	3.8	(3.3–4.5)
Female	11.9	(10.9–12.9)	4.8	(4.3–5.5)	6.4	(5.8–7.1)
Country of birth						
Australia	12.5	(11.8–13.3)	4.8	(4.3–5.3)	6.5	(5.9–7.1)
Overseas	8.2	(7.3–9.1)	2.8	(2.3–3.3)	2.4	(2.0–3.0)
Labour force status						
Employed	11.9	(11.2–12.7)	4.2	(3.8–4.8)	4.7	(4.2–5.2)
Unemployed	13.6	(10.1–18.0)	7.9	(4.7–13.1)	13.4	(9.9–17.8)
Not in the labour force	9.0	(8.1–10.1)	3.4	(2.7–4.2)	5.3	(4.6–6.2)
Education						
Post-high school	11.3	(10.5–12.1)	4.0	(3.6–4.6)	4.6	(4.2–5.1)
Year 12 only	12.9	(11.3–14.8)	5.3	(4.1–6.9)	6.9	(5.5–8.6)
Did not complete year 12	9.3	(8.0–10.8)	3.1	(2.4–3.9)	5.4	(4.6–6.4)
Marital status						
Married	8.5	(7.8–9.2)	2.3	(1.9–2.8)	2.3	(1.9–2.7)
Separated/widowed/divorced	10.8	(9.6–12.0)	4.3	(3.5–5.4)	5.8	(4.8–7.0)
Never married	15.1	(13.9–16.3)	6.6	(5.7–7.6)	9.2	(8.2–10.3)
Remoteness area (ARIA)						
Major cities	11.0	(10.3–11.8)	4.2	(3.7–4.7)	5.0	(4.5–5.5)
Inner regional	11.5	(10.1–13.0)	3.8	(2.8–5.0)	5.9	(4.8–7.1)
Outer regional/remote/very remote	10.6	(8.3–13.4)	4.0	(2.5–6.4)	5.4	(3.8–7.6)
SEIFA						
1 (Most disadvantaged)	11.0	(9.6–12.6)	4.2	(3.3–5.3)	5.6	(4.6–6.9)
2	11.0	(9.6–12.6)	3.7	(2.8–4.9)	6.5	(5.3–8.0)
3	11.7	(10.5–13.0)	4.2	(3.4–5.1)	4.3	(3.5–5.2)
4	10.1	(8.9–11.5)	4.7	(3.7–5.8)	5.0	(4.2–5.9)
5 (Least disadvantaged)	11.7	(10.4–13.2)	3.5	(2.8–4.4)	4.6	(3.7–5.6)
Psychological distress						
High psychological distress	19.4	(15.5–24.1)	13.5	(9.6–18.6)	42.0	(36.8–47.3)
Low psychological distress	10.7	(10.1–11.3)	3.6	(3.2–4.0)	3.3	(3.0–3.7)
Service use (any mental health care)						
Yes	21.0	(19.4–22.7)	13.1	(11.5–14.9)	20.2	(18.3–22.2)
No	8.9	(8.3–9.6)	2.2	(1.9–2.5)	2.0	(1.7–2.4)
Suicidality						
Yes	22.2	(16.7–28.9)	14.6	(10.6–19.8)	34.4	(28.9–40.3)
No	10.7	(10.1–11.2)	3.7	(3.4–4.1)	4.2	(3.8–4.6)

a There were a small number of cases who indicated another term for sex at birth other than male or female; however, these cases were excluded from the current analysis due to Australian Bureau of Statistics data safety rules.

**Table 4.** Multivariable multinomial logistic regression comparing demographic and clinical correlates by comorbidity groups.

	Two disorders vs one disorder		Three or more disorders vs one disorder	
	aOR^a^	95% CI	aOR^a^	95% CI
Age				
16–24	1.48	(0.97–2.27)	2.12	(1.52–2.95)
25–44	1.10	(0.76–1.58)	1.61	(1.12–2.33)
45–64	0.93	(0.62–1.40)	1.28	(0.93–1.75)
65+	1.00	(1.00–1.00)	1.00	(1.00–1.00)
Sex at birth^b^				
Female	1.27	(0.99–1.62)	1.45	(1.15–1.84)
Male	1.00	(1.00–1.00)	1.00	(1.00–1.00)
Employment				
Employed	1.00	(1.00–1.00)	1.00	(1.00–1.00)
Unemployed	1.61	(0.84–3.09)	2.51	(1.56–4.06)
Not in the labour force	1.12	(0.78–1.61)	1.91	(1.43–2.55)
Country at birth				
Australia	1.06	(0.85–1.31)	1.62	(1.28–2.05)
Other	1.00	(1.00–1.00)	1.00	(1.00–1.00)
Education				
Post-high school	1.00	(1.00–1.00)	1.00	(1.00–1.00)
Year 12 only	1.01	(0.70–1.45)	1.15	(0.81–1.64)
Did not complete year 12	0.85	(0.60–1.21)	1.45	(1.10–1.90)
Marital status				
Married	1.00	(1.00–1.00)	1.00	(1.00–1.00)
Separated/Widowed/Divorced	1.46	(1.03–2.07)	2.04	(1.45–2.87)
Never Married	1.44	(1.03–2.01)	2.13	(1.64–2.77)
Remoteness area (ARIA)				
Major cities	1.00	(1.00–1.00)	1.00	(1.00–1.00)
Inner regional	0.88	(0.62–1.25)	1.17	(0.89–1.53)
Outer regional/remote/very remote	1.05	(0.58–1.93)	1.20	(0.76–1.88)
SEIFA				
1 (Most disadvantaged)	1.22	(0.84–1.78)	1.29	(0.94–1.76)
2	1.13	(0.71–1.79)	1.58	(1.12–2.21)
3	1.18	(0.83–1.67)	0.94	(0.68–1.30)
4	1.53	(1.03–2.29)	1.27	(0.89–1.81)
5 (Least disadvantaged)	1.00	(1.00–1.00)	1.00	(1.00–1.00)
Psychological distress				
High psychological distress	2.05	(1.26–3.35)	7.25	(5.31–9.90)
Low psychological distress	1.00	(1.00–1.00)	1.00	(1.00–1.00)
Service use (any mental health care)				
Yes	2.55	(2.00–3.24)	4.24	(3.36–5.35)
No	1.00	(1.00–1.00)	1.00	(1.00–1.00)
Suicidality				
Yes	1.87	(1.14–3.04)	3.96	(2.73–5.74)
No	1.00	(1.00–1.00)	1.00	(1.00–1.00)

a Odds ratio adjusted for sex and age.

b There were a small number of cases who indicated another term for sex at birth other than male or female, however these cases were excluded from the current analysis due to Australian Bureau of Statistics data safety rules.

### Comparison with 2007 Australian NSMHWB

Overall, there was little evidence that the prevalence of any co-occurrence among those with past 12-month mental disorders changed across the surveys with 42.3% (95% CI = 38.7–45.9%) in 2007 and 45.5% (95% CI = 43.5–47.5%) in 2020–2022 (PR = 1.08, 95% CI = 0.98–1.18). There was little evidence of a significant interaction between survey year and sex at birth (PR = 0.94, 95% CI = 0.76–1.17). However, there was evidence of a significant interaction between survey year and age (Likelihood ratio test = 14.26, *p* = 0.005), suggesting that the difference in the prevalence of any co-occurrence between the survey years differed depending on age group. Further subgroup analysis indicated that the youngest age group (16–24) experienced a 44% increase in the rate of any co-occurrence in 2020/2022 in comparison to 2007 (36.6% vs 52.8%; PR = 1.44, 95% CI = 1.17–1.77), whereas there was little evidence of a significant difference for the other age groups (24–44 years: PR = 1.04, 95% CI = 0.89–1.19; 45–64 years: PR = 0.90, 95% CI = 0.74–1.08; 65+ years: PR = 1.23, 95% CI = 0.87–1.74). Finally, the CDIR values in 2007 are provided in Figure 3 (right panel). There was some consistency across the surveys in the top three disorders associated with co-occurrence, being dysthymia, bipolar and OCD with CDIR values between 2.7 and 2.9.

## Discussion

Co-occurrence of mental and substance use disorders remains a significant problem for the Australian population with 46% of people with a past 12-month mental or substance use disorder in 2020–2022 experiencing two or more diagnosable conditions. Those in the population with greater odds of experiencing three or more mental disorders in the past 12 months were female, aged 16–24 years, unemployed or not in the labour force, did not complete high school, never married or divorced/separated/widowed. Moreover, those with higher levels of psychological distress, higher service use and higher rates of suicidality were at greater odds of experiencing co-occurring disorders, with dose–response relationships appearing between number of co-occurring disorders and the experience of distress, service use and suicidality (the cross-sectional design precludes the determination of causality or directionality for these associations). In terms of patterns, the most common combination of disorders was multiple anxiety disorders followed by multiple anxiety and/or mood disorders. Specifically, those with bipolar disorder, obsessive compulsive disorder or dysthymia demonstrated higher rates of co-occurring disorders than the other analysed conditions. Overall, the experience of co-occurring disorders is endemic.

The prevalence of co-occurring disorders in Australia has not declined since the previous survey conducted in 2007. Despite some growth of research and treatment/training programmes addressing co-occurring conditions, there is a continued need for additional research to explore potential reasons for why rates of co-occurring conditions have remained high or whether the profile of factors that drive co-occurring conditions in the past have shifted. Indeed, the moderation analysis further suggests that those aged 16–24 years demonstrated increases in rates of co-occurrence with a 44% increase in the rate of any co-occurrence between 2007 and 2020/2022. This trend corresponds to previous evidence from Australia and internationally that demonstrate increases in internalising and emotional disorders among young people and more recent cohorts of young people and the increased need for early intervention prior to the onset of secondary disorders (Halladay et al., 2023; Keyes et al., 2020). As such, it appears that the prevalence of co-occurring disorders is changing among young Australians at a faster rate than older age groups and the potential environmental/social mechanisms and explanatory factors leading to increased co-occurring conditions may be changing as well. These potential cohort, age and period effects require further examination to aid in the development and evolution of services that target co-occurring mental disorders.

The impact of the global coronavirus (COVID-19) pandemic cannot be ignored and may explain the observed lack of change in the population as a whole and overall increase in rates of past 12-month co-occurrence during 2020/2022 among young people. Additional evidence has demonstrated that the pandemic was associated with a significant increase in uncertainty, life disruption, unemployment and loss of income, anxiety and psychological distress (Klein et al., 2023; Rahman et al., 2020). Furthermore, there is some evidence from surveys of young people seeking mental health services that approximately three-quarters felt their mental health was a little to a lot worse since the outbreak of the pandemic (Bell et al., 2023). More recently, two large systematic reviews have identified small to moderate increases in the prevalence of anxiety and depression over the pandemic, particularly for women, adolescents, pregnant and postpartum people, and those hospitalised with COVID-19 (Bower et al., 2023; Sun et al., 2023). However, longitudinal evidence has suggested that more recent cohorts of young people have exhibited increasing rates of emotional concerns and psychological distress, trending upwards in the years prior to the pandemic, pointing towards broader contextual and societal changes (Halladay et al., 2023; Twenge et al., 2010, 2019). Moreover, there is evidence to suggest the widening of socioeconomic differentials contributing to worsening health overall preceded the pandemic (Adair and Lopez, 2020). It remains an open question for future research to determine the potential mechanisms underlying the increased prevalence of co-occurring mental health conditions as well as the lingering effects as this younger cohort ages.

The overall pattern of co-occurrence found in the current study, as expressed in the correlation matrix provided in Figure 2, is broadly consistent with a recent large meta-analytic correlation matrix derived from 35 studies across multiple countries assessing 23 diagnoses (Ringwald et al., 2023). Increasingly, evidence suggests that the pattern of co-occurrence observed in epidemiological data might reflect a common structure that could indicate potential shared underlying mechanisms. By providing a more nuanced understanding of the underlying dimensions of psychopathology, these models may help to identify common mechanisms underlying diverse mental disorders. This, in turn, could lead to the development of more effective and efficient treatments that are tailored to individual needs and better reflect the way in which people experience these conditions.

The continuing pervasiveness of co-occurrence among mental health disorders in the Australian population, particularly among older adolescents, has several implications for prevention, early intervention and treatment. Universal prevention efforts that target broad contextual and societal factors affecting multiple mental disorders might prove beneficial at preventing the onset of co-occurring disorders. With the increasing prevalence of co-occurring disorders among young people and in more recent cohorts, it seems imperative to implement both transdiagnostic prevention and targeted early intervention programmes within school settings at an earlier age than possibly first thought (Andrews et al., 2023). Such programmes could potentially target transdiagnostic or prodromal constructs that place individuals at higher risk and are associated with increased onset of mental and substance use disorders, such as personality, early traumatic experiences, social connectedness, internalising and externalising psychopathology and other lifestyle behaviours (e.g. sleep, physical activity, diet).

A notable limitation of the current study is that conclusions regarding co-occurring disorders represent associations. The cross-sectional design precludes the ability to determine within-person changes over time, which would be required to determine causal relationships. However, a significant advantage of the current study was that consistency was maintained in the diagnostic instrument and sampling framework between the 2007 and 2020/2022 National surveys. This enables the comparison of population-level estimates in co-occurring disorders over time pointing to changes in broader contextual and/or societal factors as potential mechanisms. A second limitation is that the data were primarily self-reported, and while the diagnostic instrument was interviewer-administered, potential biases associated with retrospective recall and failure to account for clinical observation may have impacted the diagnostic data. Third, the survey received a lower than anticipated response rate of approximately 52%, as well as the lack of representation of very remote parts of Australia and discrete Aboriginal and Torres Strait Islander communities, which brings into question the overall generalisability and widespread representativeness of the sample. While this is concerning, it is of note that the observed response rate is comparable to international trends in survey-based research where response rates have been trending downward over the past 10–20 years.

This study provides the first comprehensive estimates of patterns, prevalence and correlates of co-occurring mental and substance use disorders among the Australian population in 13 years. Co-occurring mental and substance use disorders remain an endemic feature of population health. Co-occurrence of mental and substance use disorders is particularly problematic in younger more recent cohorts, where increases in both type and severity of co-occurring conditions have been observed. The findings underscore the need for improved approaches to prevention and early intervention and support recent calls for increased investment in improving the mental health of young Australians.
